# Evaluation of the temperature of posterior lower limbs skin during the whole body vibration measured by infrared thermography: Cross-sectional study analysis using linear mixed effect model

**DOI:** 10.1371/journal.pone.0212512

**Published:** 2019-03-13

**Authors:** Eloá Moreira-Marconi, Marcia Cristina Moura-Fernandes, Patrícia Lopes-Souza, Ygor Teixeira-Silva, Aline Reis-Silva, Renata Marques Marchon, Eliane de Oliveira Guedes-Aguiar, Laisa Liane Paineiras-Domingos, Danúbia da Cunha de Sá-Caputo, Danielle Soares Morel, Carla Fontoura Dionello, Sérgio Oliveira De-Carvalho, Mario José dos Santos Pereira, Arlete Francisca-Santos, Gefferson Silva-Costa, Marcio Olímpio-Souza, Tânia Regina Lemos-Santos, Nasser Ribeiro Asad, Vinicius Layter Xavier, Redha Taiar, Anelise Sonza, Adérito Seixas, Darryl J. Cochrane, Mario Bernardo-Filho

**Affiliations:** 1 Programa de Pós-Graduação em Fisiopatologia Clínica e Experimental, Universidade do Estado do Rio de Janeiro, Rio de Janeiro, Brazil; 2 Laboratório de Vibrações Mecânicas e Práticas Integrativas—LAVIMPI, Instituto Biologia Roberto Alcântara Gomes e Policlínica Américo Piquet Carneiro, Universidade do Estado do Rio de Janeiro, Rio de Janeiro, Brazil; 3 Programa de Pós-Graduação em Ciências Médicas, Universidade do Estado do Rio de Janeiro, Rio de Janeiro, Brazil; 4 Mestrado Profissional em Saúde, Medicina Laboratorial e Tecnologia Forense, Universidade do Estado do Rio de Janeiro, Rio de Janeiro, Brazil; 5 Programa de Pós-Graduação em Ciências da Saúde, Universidade Federal do Rio Grande do Norte, Rio Grande do Norte, Brazil; 6 Faculdade Bezerra de Araújo, Rio de Janeiro, Brazil; 7 Centro Universitário Serra dos Órgãos, Teresópolis, Rio de Janeiro, Brasil; 8 Departamento de Estatística, Instituto de Matemática e Estatística, Universidade do Estado do Rio de Janeiro, Rio de Janeiro, Brazil; 9 Groupe de Recherche en Sciences pour l’Ingénieur (GRESPI)/Université de Reims Champagne Ardenne, France; 10 Universidade Estadual de Santa Catarina, Florianópolis, SC, Brazil; 11 Escola Superior de Saúde, Universidade Fernando Pessoa, Porto, Portugal; 12 School of Sport, Exercise & Nutrition, Massey University, Palmerston North, New Zealand; Nottingham Trent University, UNITED KINGDOM

## Abstract

**Background:**

Whole body vibration exercise (WBVE) has been shown to improve muscular strength and power, and increase peripheral blood flow. The aim of this study was to evaluate the behavior of the skin temperature (Tsk) on regions of the lower limbs from an acute bout of WBVE.

**Methods and findings:**

Cross-sectional study approved by local ethics committee (*Certificado de Apresentação para Apreciação Ética*—*CAAE—*19826413.8.0000.5259) and Trial registration (*Registro Brasileiro de Ensaios Clínicos—REBEC*—RBR-738wng). Using Infrared thermography (IRT), Tsk and thermal symmetry of the posterior lower extremities (thigh, knee, calf and heel) were examined in 19 healthy participants. IRT was assessed during 60-second WBVE exposures of 0, 30 and 50 Hz. From the adjusted linear mixed effects model, vibration frequency, time and regions of the lower extremity were significant (*p*<0.001). However, the variable laterality was not significant (*p* = 0.067) and was excluded from the adjusted statistical model. The adjusted model was significant (*p*<0.00001) and all variables in the model were significant (*p*<0.01) indicating that Tsk decreases with time, independently of the vibration frequency. The value of the Pseudo-R-Squared for the model was 0.8376. The presented mathematical model of the current study may be useful to justify the patterns observed for all vibration frequencies between and 0 and 50 Hz. The main limitations of the study were the reduced time of the intervention and not having evaluated other regions of the body.

**Conclusions:**

The acute exposure of 60-second mechanical vibration has effect on the behavior of Tsk of the posterior region of the lower limbs, which is likely to be associated with a decrease on the blood flow due to WBVE. It is speculated that during WBVE a greater supply of blood is required where the body responds by shunting blood flow from the skin to working muscle in the first seconds of exercise. Further investigative work is required to verify this hypothesis.

## Introduction

It is well established that the skin, the largest organ of the body, is highly implicated in the thermoregulation process [1]. The human body is homoeothermic, in which thermal cutaneous signals provide feedback to the thermoregulation system, reducing its response time and making body core temperature more stable. There is evidence that the autonomic nervous system is the primary regulator of blood circulation in the skin, that acts as the main regulator of thermal emission [1]. However, the regulation of skin temperature (Tsk) depends on the rate of blood flow, structures of the subcutaneous tissue and the activity of the autonomic nervous system [2]. Change in Tsk may be an indicator of abnormalities, such as a disease [2,3]. Ring and Ammer [3] and Lahiri *et al*. [4] have reported that thermal imaging has been used to study diseases where Tsk can reflect the presence of inflammation in underlying tissues, or where blood flow is increased or decreased due to a clinical abnormality.

The use infrared thermography (IRT) is growing in popularity as a diagnostic tool for applied medicine or as outcome measure for clinical trials [5–8]. IRT allows imagining of the body surface temperature in real time with high sensitivity (up to 0.025°C) and accuracy up to 1% without physical contact [9–11]. This technology quantifies the infrared radiation emitted by a surface and converts the intensity of radiation to temperature values, allowing the monitoring of dynamical variations of temperature and is considered an emerging methodological alternative to analyzing Tsk [10]. Its use has also included the monitoring of sports training and in the assessment of the workload quantification [12–14]. Additionally, IRT has been utilized to monitor physiological reactions induced by non-pharmacological interventions such as massage [15,16], manual therapy [17,18] and physical exercise [7,19,20]. Therefore, IRT is a promising tool to detect changes in Tsk caused by exercise by monitoring body surface temperature before, during and after movement [14,20,21]. This is especially important when determining the authenticity of new exercise modalities, such as whole body vibration exercise (WBVE). For instance, there has been a growing interest in the use of WBVE. It has been suggested as a complementary modality to traditional exercise where it is acknowledged as a viable option in sport and health care [22]. However, the physiological effects of WBVE are still not fully understood and its efficacy for safe clinical application requires further research. Generally, WBVE is performed by standing on a commercially manufactured machine with an oscillating-vibratory platform (OVP) where the physiological effects are influenced by parameters such as, vibration frequency, peak-to-peak displacement, work and rest times, total exposure time, number of bouts per session, periodicity of sessions and body position [23]. The load on the neuromuscular system is imposed by biomechanical vibration parameters and according to Luo *et al*. [24] vibration frequencies between 30 Hz and 50 Hz optimize muscle activity. Moreover, performing WBVE in a squat position increases perfusion, which requires it to comply with higher muscle demands—as a result muscular activity increases to attenuate the imposed vibration. This muscular activity elicits rhythmic muscle contractions that increases peripheral circulation by increasing the diameter size of small vessels in the quadriceps and gastrocnemius [25,26]. Additionally, Lythgo *et al*. [27] demonstrated that isometric squatting alone can significantly increase leg blood flow and that higher vibration frequencies can further enhance leg blood flow.

The effect of WBVE on Tsk remains equivocal. Previous researchers have reported a significant increase [28,29], non-significant change [30,31] and a significant decrease in Tsk [32,33], however the intervention and assessment protocols have widely been heterogeneous. Moreover, there is a limited number of studies that have assessed the effect of different vibration frequencies on Tsk [32], and this would be highly relevant. Given the lack of agreement among the studies and the dearth of research comparing the effects of different vibration frequencies on Tsk, the aim of this study was to evaluate the effects of WBVE in healthy individuals exposed to a single session of 0, 30 and 50 Hz. The hypothesis is that the effect of WBVE, evaluated by the Tsk, would be related to frequency, time, laterality (right and left legs) and regions of interest (ROI) over 60-seconds. Moreover, Tsk symmetry would be similar in right and left legs.

## Materials and methods

### Study design

This was a cross-sectional study performed in a single session. The local ethics committee approved the study (*Certificado de Apresentação para Apreciação Ética*—*CAAE—*19826413.8.0000.5259) and Trial registration (*Registro Brasileiro de Ensaios Clínicos—REBEC*—RBR-738wng) and it was conducted in accordance with the Resolution 158 N 466/12 of the National Health Council and the Declaration of Helsinki. The protocol was deposited in “protocols.io” (dx.doi.org/10.17504/protocols.io.wqhfdt6). All volunteers gave written informed consent to participate in the work. To detect a before-and-after difference in lower limb temperature of 1.5 ± 0.9 °C, with a two-sided 5% significance level at a power of 90%, with an anticipated dropout rate of 10%, a minimum sample size of 19 participants was required [34].

### Participants

**Fig 1:**
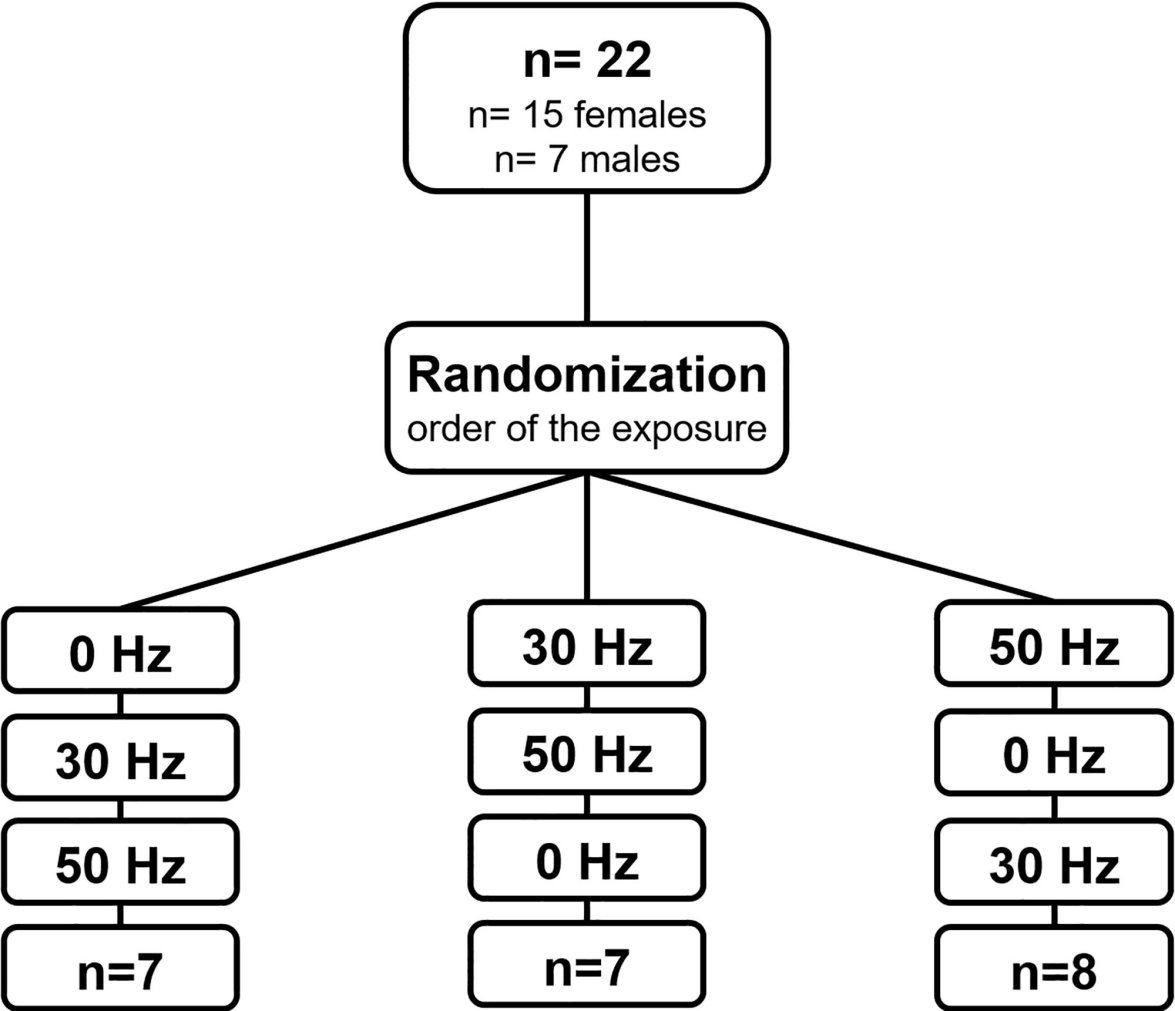
Flowchart.

Nineteen participants (physiotherapists, physicians, biologists) volunteered for the study (15 females and 7 males). The participants had to meet the following inclusion criteria of aged 20 to 45 years old and healthy. The exclusion criteria were; joint pain and/or implants, musculoskeletal diseases, vertigo and other clinical diseases that could involve some risk or discomfort during WBVE, other declared disease. To reduce bias the order of the exposure was randomized using colored cards (Fig 1), and those assessing the outcomes and the participants do not know order of the frequencies.

### Intervention

The intervention was performed in the *Laboratório de Vibrações Mecânicas e Práticas Integrativas (LAVIMPI)*, *Universidade do Estado do Rio de Janeiro (UERJ)* at December 27th, 2017 with controlled ambient temperature (25.5 ± 0.35°C) and relative humidity (50.5 ± 2.16%). All procedures were followed by health professionals (physicians, physiotherapists and biologists). Before the intervention, all participants were provided with a 15-minute acclimation period to ensure Tsk was stabilized. To avoid any effect of circadian rhythm participants performed the intervention session between 1–5 pm. A health-activity interview about the caffeine intake, smoking (nicotine) and sports training, at least, in the previous four hours before the intervention was conducted. The measurement of body mass and height and the determination of the body mass index were carried out [35] prior to the intervention. During the intervention, participants wore shorts, t-shirt and were barefooted.

**Fig 2:**
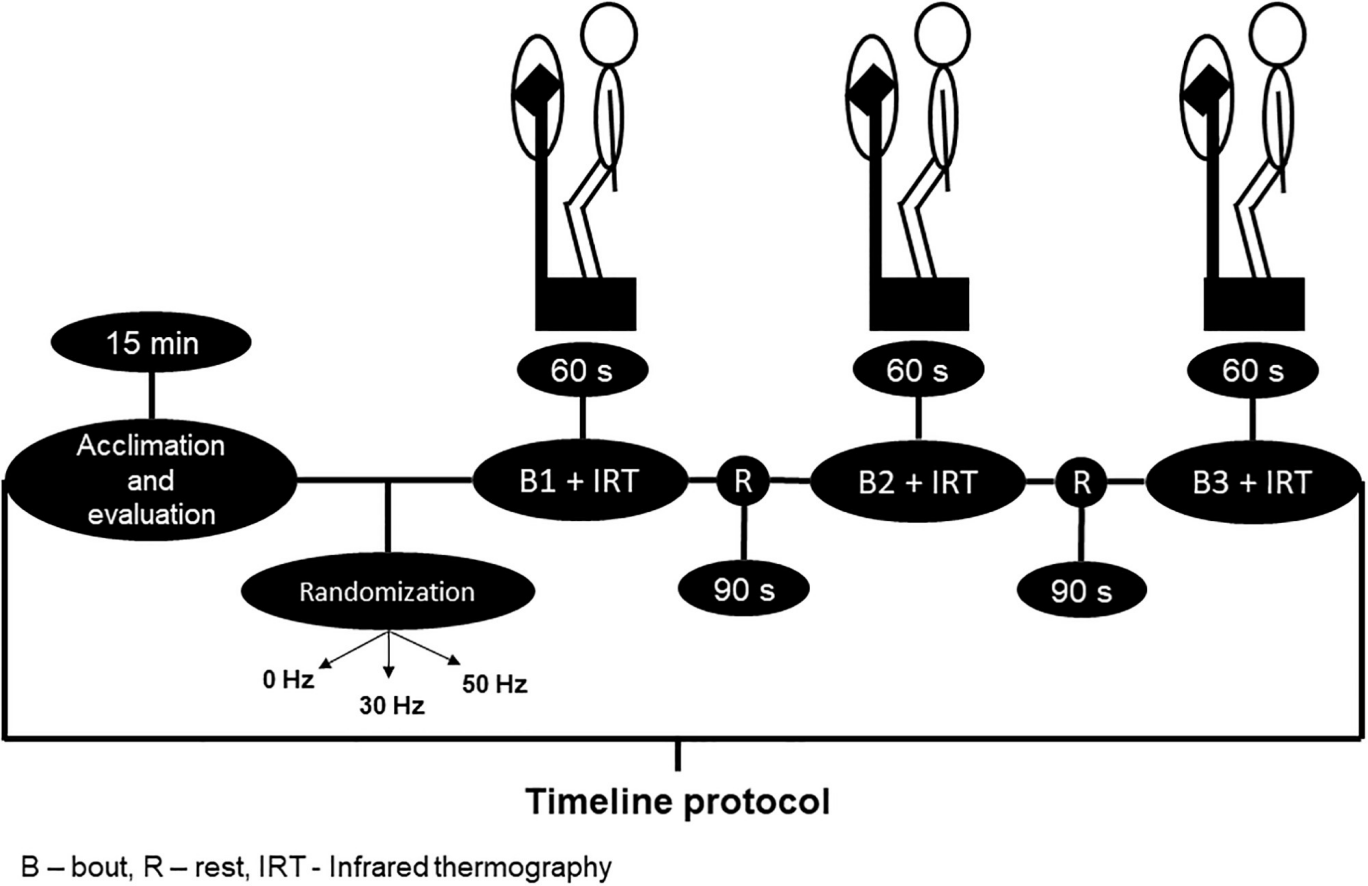
Whole body vibration exercise intervention.

The acute intervention was performed on a commercial machine (Power Plate pro5 ^™^, Power Plate International Ltd, The Netherlands) that had a vertical tri-planar OVP. The intervention consisted of a single session with three bouts of 60-seconds with 90-seconds rest separating each bout. Participants assumed a squat position on the base of the OVP with 130° knee flexion (measured by a manual goniometer), the distance of the feet followed the line of the shoulders and the arms to the side (Fig 2). Participants were exposed to three experimental protocols of squat position + without vibration (0 Hz); squat position + WBVE 30 Hz (frequency), 1.20 mm (amplitude) and squat position + WBVE 50 Hz (frequency), 0.77 mm (amplitude).

A three-axial accelerometer (Vibration Datalogger DT-178A, Ruby Electronics, Saratoga, USA) was fixed on the base of the OVP to verify the peak acceleration and gravitational force of the OVP. The vibration frequency of 30 and 50 Hz with 1.20 and 0.77 mm of amplitude produced a gravitational force of 2.22 *g* and 4.40 *g* at a peak acceleration of 21.73 m/s^2^ and 43.11 m/s^2^, respectively.

#### Lower limb temperature assessment

Skin temperature was assessed using IRT and data collection followed the recommendations of previously published studies [36,37]. An infrared camera (FLIR Systems, E40, Wilsonville, OR, USA), with a sensor array size of 160 x 120 pixels and noise equivalent temperature difference (NETD) of 70mK at 30°C and had ±2% repeatability of the overall reading. The camera’s emissivity was set to 0.98 and images were captured and processed using the software FLIR ResearchIR Max (version 4.40.4.17, Sweden).

The camera was placed at an angle of approximately 90° to the surface and 2 m from the WBVE machine to provide a full view of the lower limbs. The height of the camera varied according to the height of each participant corresponding to 10 cm below the popliteal line of the right leg of each individual.

Fig 2 also illustrates the sequence of the different steps of the various procedures starting with the acclimation of the participants up to the acquisition of the IRT, as presented in the timeline.

**Fig 3:**
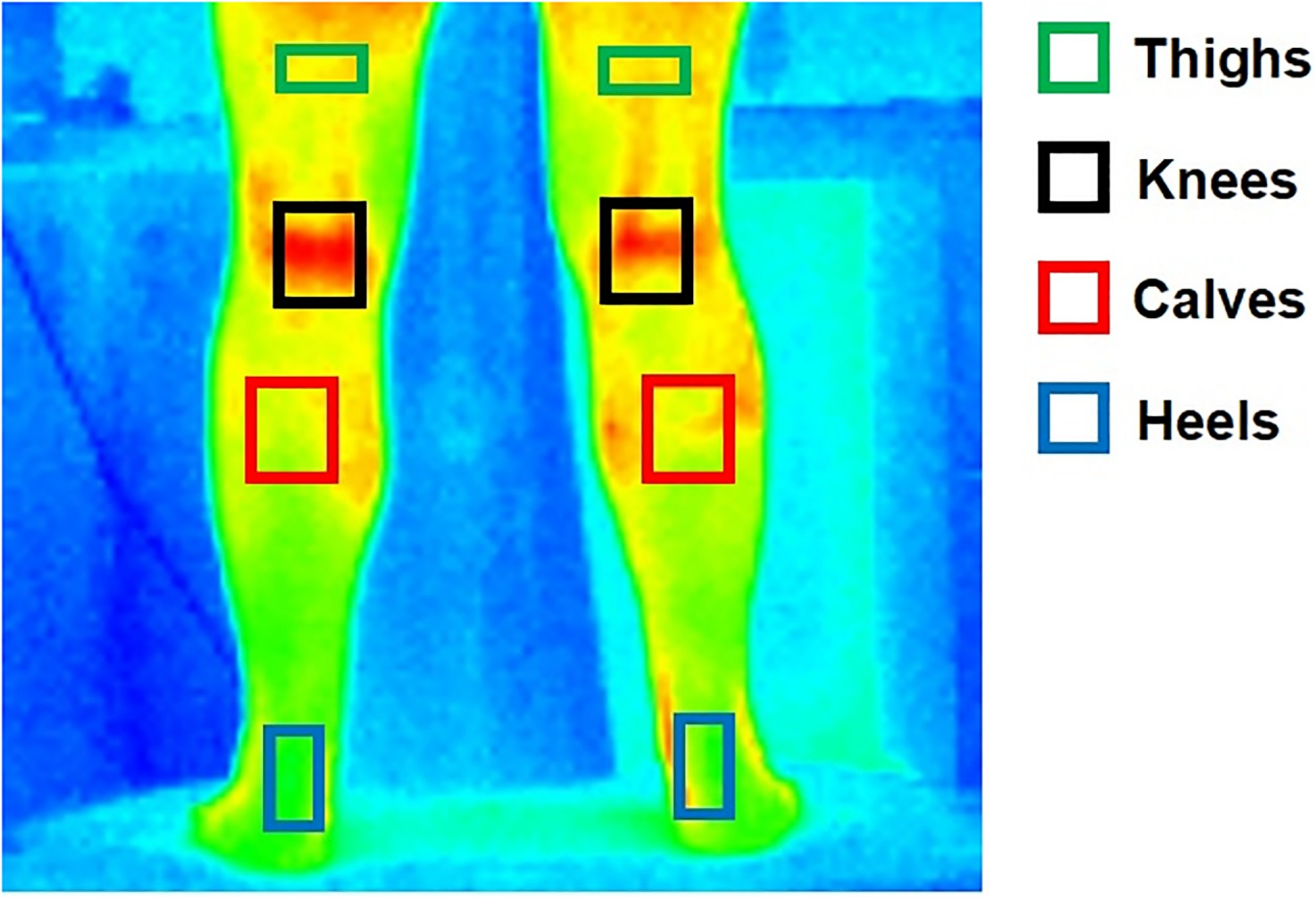
Regions of interest (ROI).

A sequence of images of the posterior aspect of the lower limbs was automatically recorded for 1 minute in each bout at 30 frames per second. These 1800 images were divided in five equal ranges with 360 frames each. The average of the area selected, consisted of 12 seconds of duration, were obtained to represent the mean time of range (6, 18, 30, 42, and 54 seconds, respectively). In all time frames a focal line was used to assess the same ROI. Fig 3 shows the ROI used in the analysis (left and right thighs, left and right knees, left and right calves, left and right heels).

### Statistical analysis

A linear mixed effects model was adjusted using temperature as a dependent variable. Due to the repeated measures for each participant and individual difference in basal body temperature, the participants were set as the random effect component in the model. The fixed effect included the variables of frequency, time, laterality and ROI. The categorical variable ROI had four levels (thighs, knees, calves and heels). The level of the heels was set as the baseline due it having the lowest temperature. The regression was performed with the intention to estimate the effect of frequency, time, laterality and ROI in Tsk. All statistical analysis was performed using R software, version 3.5.0 (R Foundation of Statistical Computing, Vienna, Austria) and the R libraries [38,39]. Results are considered statistically significant If the p-value is under 0.01.

## Results

All the individuals declared, in the health-activity interview, no caffeine intake, no smoking (nicotine) and no sports training, at least, in the previous four hours before the intervention. The characteristics of the participants were mean (± standard deviation) age 37.0 ± 4.24 year; height 1.66 ± 0.04 m; body mass 73.49 ± 0.64 kg; body mass index 26.6 ± 1.48 kg/m^2^. All the individuals included in this study, concluded it.

**Fig 4:**
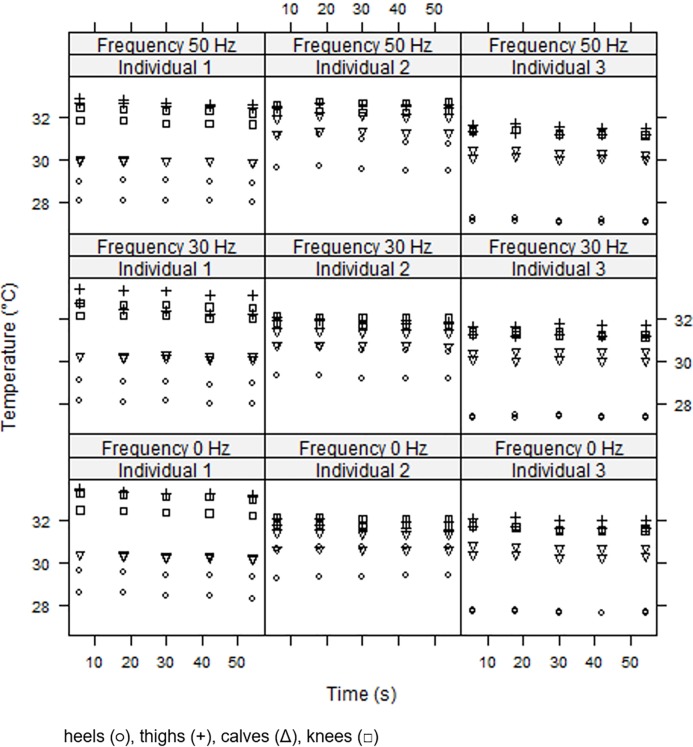
Illustration of the effect of whole body vibration exercise (three individuals) on skin temperature of the posterior area of the lower limbs.

Fig 4 illustrates the pattern of the variation, in three individuals, of Tsk in different regions of interest on the right and on the left lower limbs (heels, thighs, knees and calves) with the exposure time at three vibration frequencies (0, 30 and 50 Hz) of WBVE. In almost of all participants, Tsk decreased with time, independently of the tested vibration frequency.

In the adjusted linear mixed effects model, variables of vibration frequency, time and ROI were significant (*p*<0.001), however the variable laterality was not significant (*p* = 0.067). Thus, this variable was excluded of the model and a new model was adjusted. The F-test of overall significance determines whether the regression model is statistically significant and the Pseudo-R-Squared is a statistical measure of how close the data are to the fitted regression line. Table 1 shows the result of the linear mixed effects model without the variable laterality. The adjusted model was significant (F-test, *p*<0.00001) and all variables in the model were significant (Intercept, Time, Thighs, Calves and Knees, *p*<0.001, Frequency *p*<0.01). The value of the Pseudo-R-Squared for this model was 0.8376.

**Table 1:** The linear mixed effects model—dependent variable Tsk.

Variable	Coefficient	*p-value*
Intercept	28.588	<0.0001
Frequency (Hz)	-0.002	0.0036
Time (s)	-0.003	<0.0001
thighs	2.841	<0.0001
calves	1.994	<0.0001
knees	3.022	<0.0001

Pseudo-R-Squared = 0.8376

The intercept (28.588) represents the standard temperature of the heels, which was set as the baseline. To estimate the mean temperature for each location considering a specific vibration frequency and a specific time, add the value of the coefficient intercept (28.588) to the value of the coefficient of the location (thighs = 2.841, calves = 1.994, knees = 3.022) plus the specific frequency multiplied by the coefficient frequency (-0.002) plus the specific time multiplied by the coefficient time (-0.003), according to the following regression model equation:

Tsk=28.588+Coefficientofthelocation+Frequency.(-0.002)+Time.(-0.003)

## Discussion

The purpose of this study was to evaluate the behavior of Tsk on WBVE using IRT. We hypothesized Tsk symmetry would be similar in right and left legs, which was confirmed by our results. The autonomic nervous system controls the blood flow of the skin so that it is anatomically and histologically symmetrical [40,41]. This symmetry can also occur in Tsk. The difference between the contralateral ROI tends to be minimal [40,41]. This fact may be responsible for the similar results in the right and left lower limbs, with laterality not being a significant variable in the adjusted statistical model. Chudecka *et al*, [42] have reported that in the scullers, the mean Tsk was always lower post than pre exercise, with no significant differences in an average temperature drop between the opposite sides, indicating that the work of the muscles involved in the physical exertion on the rowing ergometer was symmetrical. In addition, in the handball players, the Tsk in symmetric areas over the asymmetrically working muscles showed statistically significant differences between sides, which was associated with the functional asymmetry of training. Selfe *et al*. [43] reported that a difference greater than 1°C between body symmetry may indicate pathophysiological manifestation. This illustrates that thermal symmetry between limbs is essential in evaluating uniformity. The current result suggests that the oscillating nature of WBVE was not detrimental to participants in promoting a negative response. Due to the action of the mechanical vibration, WBVE is associated with higher muscle activity [44,45], increased motor unit recruitment [46], enhanced blood flow [47], increased cutaneous sensitivity [48], and improved balance [44,49]. Moreover, this finding is in agreement with the results of Chudecka *et al*. [42] with scullers.

In the present study, Tsk in the posterior region decreased in all experimental conditions (*p*<0.001). The Tsk variation pattern was similar in all conditions, corresponding to a Tsk decrease, independently of the vibration frequency. This observation may be explained by the fact that WBVE initiates a rapidly and repeating eccentric-concentric action that evokes muscular work and elevates the body’s metabolic rate [50] increasing in intramuscular temperature [31]. This modifies the thermal balance that activates the mechanism responsible for heat loss, which is mediated by the hypothalamic feedback system [51]. This change causes a redistribution of blood flow from inactive to active areas. Subsequently, with the continuation of WBVE the redirection of blood flow to the skin occurs, allowing heat exchange with the environment [52,53]. This process aims to cool the skin so that the blood that is perfused returns to the internal body regions at a lower temperature, thus avoiding a dangerous state of hyperthermia [54]. Another plausible explanation is that mechanical vibration induces a vasoconstrictor response to decrease Tsk [34]. The present results are in agreement with Seixas *et al*. [32] who reported a significant decrease in Tsk after 5-minutes of WBVE (frequency = 35 and 45 Hz, amplitude = 5–6 mm) of the anterior thighs, that included lateral and medial areas. In contrast, Cochrane *et al*. [31] and Cochrane *et al*. [30] observed non-significant Tsk change, however [28], and Hazel *et al*. [29] reported a significant Tsk increase. It is important to note that the interventions and assessment protocols were heterogeneous, which may explain the discrepancies among the studies.

Seixas *et al*, [32] reported no significant difference in Tsk from WBVE, however it was not documented that there was a significant difference in Tsk between the groups before the commencement of WBVE. Together with the small sample size of each group (n = 6) and the small increment in vibration frequency (5 Hz) may have led to the non-significant finding. Although no significant differences in Tsk after the exposure to WBVE were reported, the study examined 20 ROI (in both lower limbs) and in the group exposed to 35 Hz Tsk significantly decreased in only 6 ROI while in the group exposed to 40 Hz Tsk significantly decreased in all ROI. In addition, Seixas *et al*. [32] study did not evaluate the temporal behavior of WBVE. In contrast, the current findings revealed that both 30 Hz and 50 Hz significantly decreased Tsk in calves, thighs and knees. A plausible explanation is that during WBVE a greater supply of blood is required in the active musculature where the body responds by shunting blood flow from the skin to working muscle in the first seconds of the 60-second exercise bout. In support of the current findings Sonza *et al*, [34] observed a decrease in Tsk of the lower limb temperature during 15 minutes and 10 minutes following WBVE of different vibration frequencies (31, 35, 40 and 44 Hz).

The presented mathematical model of the current study may be useful to justify the patterns observed for all vibration frequencies between and 0 and 50 Hz. Furthermore, this statement can justify and is in agreement with the results obtained by Sonza *et al*. [34] that have observed a decrease of Tsk of both legs due to WBVE in frequencies of the interval from 31 up to 44 Hz. The main limitations of the study were the reduced time of the intervention and not having evaluated other regions of the body.

## Conclusion

In conclusion, acute exposure of 60-second mechanical vibration has effect on the behavior of Tsk of the posterior region of the lower limbs, which is likely to be associated with a decrease on the blood flow due to WBVE. It is speculated that during WBVE a greater supply of blood is required where the body responds by shunting blood flow from the skin to working muscle in the first seconds of exercise. Further investigative work is required to verify this hypothesis.

## Supporting information

S1 Fig Trial registration (*Registro Brasileiro de Ensaios Clínicos—REBEC*—RBR-738wng). (PDF)

S2 Fig Trend statement (TREND) checklist. (PDF)

## References

[1] Charkoudian N. Skin blood flow in adult human thermoregulation: How it works, when it does not, and why. Mayo Clinic Proceedings. 2003; 78(5):603–612. 10.4065/78.5.603 12744548

[2] Romanovsky AA. Skin temperature: Its role in thermoregulation. Acta Physiologica. 2014;210(3):498–507. 10.1111/apha.12231 24716231 PMC4159593

[3] Ring EFJ, Ammer K. Infrared thermal imaging in medicine. Physiol Meas Physiol Meas. 2012;33: 33–46. 10.1088/0967-3334/33/3/R33 22370242

[4] Lahiri BB, Bagavathiappan S, Jayakumar T, Philip J. Medical applications of infrared thermography: A review. Infrared Physics and Technology. 2012; 55(2012) 221–235. 10.1016/j.infrared.2012.03.007 PMC7110787 32288544

[5] Sivanandam S, Anburajan M, Venkatraman B, Menaka M, Sharath D. Medical thermography: A diagnostic approach for type 2 diabetes based on non-contact infrared thermal imaging. Endocrine. 2012;42: 343–351. 10.1007/s12020-012-9645-8 22411072

[6] Skorupska E, Rychlik M, Pawelec W, Bednarek A, Samborski W. Trigger point-related sympathetic nerve activity in chronic sciatic leg pain: a case study. Acupunct Med. 2014;32: 418–422. 10.1136/acupmed-2013-010504 24970043

[7] Fernandes A de A, Amorim PR dos S, Brito CJ, Sillero-Quintana M, Marins JCB. Regional skin temperature response to moderate aerobic exercise measured by infrared thermography. Asian J Sports Med. 2016;7(1): e29243 10.5812/asjsm.29243 27217931 PMC4870826

[8] Magalhaes C, Vardasca R, Mendes J. Recent use of medical infrared thermography in skin neoplasms. Skin Research and Technology. 2018;24(4):587–591. 10.1111/srt.12469 29575378

[9] Andal R, Deporte M, Espa T. Variation of skin temperature during and after contrast bath therapy. Rev Andal Med Deport. 2011; 4(4):129–134.

[10] Vargas JVC, Brioschi ML, Dias FG, Parolin MB, Mulinari-Brenner FA, Ordonez JC, et al Normalized methodology for medical infrared imaging. Infrared Phys Technol. 2009;52(1): 42–47. 10.1016/j.infrared.2008.11.003

[11] Luhmann T, Piechel J, Roelfs T. Geometric calibration of thermographic cameras. Remote Sensing and Digital Image Processing. 2013 17:27–42. 10.1007/978-94-007-6639-6_2

[12] Brukner & Khan’s Clinical Sports Medicine, 4th ed Brukner Peter, Karim Khan Sydney: McGraw-Hill Australia; 2012

[13] Neves EB, Reis VM. Fundamentos da Termografia para o Acompanhamento do Treinamento Desportivo. Rev UNIANDRADE. 2014;15: 79–86. 10.18024/1519-5694/revuniandrade.v15n2p79-86

[14] Ferreira JJA, Mendonça LCS, Nunes LAO, Andrade Filho ACC, Rebelatto JR, Salvini TF. Exercise-associated thermographic changes in young and elderly subjects. Ann Biomed Eng. 2008;36:1420–1427. 10.1007/s10439-008-9512-1 18470619

[15] Sefton JM, Yarar C, Berry JW, Pascoe DD. Therapeutic Massage of the Neck and Shoulders Produces Changes in Peripheral Blood Flow When Assessed with Dynamic Infrared Thermography. J Altern Complement Med. 2010; 16(7):723–732. 10.1089/acm.2009.0441 20590481

[16] Holey LA, Dixon J, Selfe J. An exploratory thermographic investigation of the effects of connective tissue massage on autonomic function. J Manipulative Physiol Ther. National University of Health Sciences; 2011;34:457–462. 10.1016/j.jmpt.2011.05.012 21875520

[17] Roy RA, Boucher JP, Comtois AS. Paraspinal Cutaneous Temperature Modification After Spinal Manipulation at L5. J Manipulative Physiol Ther. National University of Health Sciences; 2010;33:308–314. 10.1016/j.jmpt.2010.03.001 20534318

[18] Wu CL, Yu KL, Chuang HY, Huang MH, Chen TW, Chen CH. The Application of Infrared Thermography in the Assessment of Patients With Coccygodynia Before and After Manual Therapy Combined With Diathermy. J Manipulative Physiol Ther. National University of Health Sciences; 2009;32:287–293. 10.1016/j.jmpt.2009.03.002 19447265

[19] Escamilla-Galindo VL, Estal-Martínez A, Adamczyk JG, Brito CJ, Arnaiz-Lastras J, Sillero-Quintana M. Skin temperature response to unilateral training measured with infrared thermography. J Exerc Rehabil. 2017;13:526–534. 10.12965/jer.1735046.523 29114526 PMC5667598

[20] Merla A, Mattei PA, Di Donato L, Romani GL. Thermal imaging of cutaneous temperature modifications in runners during graded exercise. Ann Biomed Eng. 2010;38:158–163. 10.1007/s10439-009-9809-8 19798579

[21] Hardaker NJ, Moss AD, Richards J, Jarvis S, McEwan I, Selfe J. The relationship between skin surface temperature measured via Non-contact thermal imaging and intra-muscular temperature of the Rectus Femoris muscle. Thermol Int. 2007;17:45–50.

[22] Cochrane DJ. Vibration exercise: The potential benefits. Int J Sports Med. 2011;32: 75–99. 10.1055/s-0030-1268010 21165804

[23] Rauch F, Sievanen H, Boonen S, Cardinale M, Degens H, Felsenberg D, et al Reporting whole-body vibration intervention studies: Recommendations of the International Society of Musculoskeletal and Neuronal Interactions. J Musculoskelet Neuronal Interact. 2010;10(3):193–198. 20811143

[24] Luo J, McNamara B, Moran K. The use of vibration training to enhance muscle strength and power. Sports Medicine. 2005;35(1):23–41. 10.2165/00007256-200535010-00003 15651911

[25] Rittweger J. Vibration as an exercise modality: How it may work, and what its potential might be. European Journal of Applied Physiology. 2010;108(5):877–904. 10.1007/s00421-009-1303-3 20012646

[26] Kerschan-Schindl K, Grampp S, Henk C, Resch H, Preisinger E, Fialka-Moser V, et al Whole-body vibration exercise leads to alterations in muscle blood volume. Clin Physiol. 2001;21:377–382. 10.1046/j.1365-2281.2001.00335.x 11380538

[27] Lythgo N, Eser P, De Groot P, Galea M. Whole-body vibration dosage alters leg blood flow. Clin Physiol Funct Imaging. 2009;29:53–59. 10.1111/j.1475-097X.2008.00834.x 19125731

[28] Games KE, Sefton JM. Whole-body vibration influences lower extremity circulatory and neurological function. Scand J Med Sci Sport. 2013;23: 516–523. 10.1111/j.1600-0838.2011.01419.x 22107331

[29] Hazell TJ, Thomas GWR, DeGuire JR, Lemon PWR. Vertical whole-body vibration does not increase cardiovascular stress to static semi-squat exercise. Eur J Appl Physiol. 2008;104:903–908. 10.1007/s00421-008-0847-y 18712407

[30] Cochrane DJ, Stannard SR, Firth EC, Rittweger J. Comparing muscle temperature during static and dynamic squatting with and without whole-body vibration. Clin Physiol Funct Imaging. 2010;30:223–229. 10.1111/j.1475-097X.2010.00931.x 20491843

[31] Cochrane DJ, Stannard SR, Sargeant AJ, Rittweger J. The rate of muscle temperature increase during acute whole-body vibration exercise. Eur J Appl Physiol. 2008;103:441–448. 10.1007/s00421-008-0736-4 18392845

[32] Seixas A, Vardasca R, Gabriel J. The Effect of Different Vibration Frequencies in the Skin Temperature in Healthy Subjects. 2014 IEEE Int Symp Med Meas Appl. 2014;1–5. 10.1109/MeMeA.2014.6860150

[33] Seixas A, Silva A, Gabriel J, Vardasca R. The effect of whole-body vibration in the skin temperature of lower extremities in healthy subjects. Thermol Int. 2012;22:59–66.

[34] Sonza A, Robinson CC, Achaval M, Zaro MA. Whole body vibration at different exposure frequencies: Infrared thermography and physiological effects. Sci World J. Hindawi Publishing Corporation. 2015;2015:452657 10.1155/2015/452657 25664338 PMC4310482

[35] World Health Organisation (WHO). BMI classification [Internet]. [cited 27 May 2018]. http://apps.who.int/bmi/index.jsp?introPage=intro_3.html

[36] Schwartz RG, Elliott R, Goldberg GS, Govindan S, Conwell T, Hoekstra PP, et al Guidelines for neuromusculoskeletal thermography. Thermology International. 2006;16(1):5–9.

[37] Moreira DG, Costello JT, Brito CJ, Adamczyk JG, Ammer K, Bach AJEE, et al Thermographic imaging in sports and exercise medicine: A Delphi study and consensus statement on the measurement of human skin temperature. J Therm Biol. 2017;69:155–162. 10.1016/j.jtherbio.2017.07.006 29037377

[38] Pinheiro J, Bates D, DebRoy S, Sarkar D, R Core Team. Linear and Nonlinear Mixed Effects Models [R package nlme version 3.1–137]. Comprehensive R Archive Network (CRAN); https://cran.r-project.org/web/packages/nlme/index.html

[39] Bartoń K. MuMIn: Multi-Model Inference [Internet]. 2018 [cited 30 Aug 2018]. https://cran.r-project.org/web/packages/MuMIn/index.html

[40] Vardasca R, Ring F, Plassmann P, Jones C, Ring EFJ, Plassmann P, et al Thermal symmetry of the upper and lower extremities in healthy subjects. Thermology International. 2012; 22(2):53–60.

[41] Niu HH, Lui PW, Hu JS, Ting CK, Yin YC, Lo YL, et al Thermal symmetry of skin temperature: normative data of normal subjects in Taiwan. Zhonghua Yi Xue Za Zhi. 2001;64: 459–68. http://www.ncbi.nlm.nih.gov/pubmed/11720145 11720145

[42] Chudecka M, Lubkowska A, Leźnicka K, Krupecki K. The use of thermal imaging in the evaluation of the symmetry of muscle activity in various types of exercises (Symmetrical and Asymmetrical). J Hum Kinet. 2015;49: 141–147. 10.1515/hukin-2015-0116 26839614 PMC4723162

[43] Selfe J, Whitaker J, Hardaker N. A narrative literature review identifying the minimum clinically important difference for skin temperature asymmetry at the knee. Thermology International. 2008; 18(2):51–54.

[44] Kim Y-Y, Park S-E. Comparison of whole-body vibration exercise and plyometric exercise to improve isokinetic muscular strength, jumping performance and balance of female volleyball players. J Phys Ther Sci. 2016;28:3140–3144. 10.1589/jpts.28.3140 27942136 PMC5140816

[45] Yu CH, Kang SR, Kwon TK. Fundamental study of lower limb muscle activity using an angled whole body vibration exercise instrument. Biomed Mater Eng. 2014;24: 2437–2445. 10.3233/BME-141057 25226944

[46] Dionello CFCF, De Souza PLPL, Sá-Caputo D, Morel DSDS, Moreira-Marconi E, Paineiras-Domingos LLLL, et al Do whole body vibration exercises affect lower limbs neuromuscular activity in populations with a medical condition? A systematic review. Restorative Neurology and Neuroscience. 2017;35(6):667–681. 10.3233/RNN-170765 29172012

[47] Button C, Anderson N, Bradford C, Cotter JD, Ainslie PN. The effect of multidirectional mechanical vibration on peripheral circulation of humans. Clin Physiol Funct Imaging. 2007; 27(4):211–216. 10.1111/j.1475-097X.2007.00739.x 17564669

[48] Sonza A, Maurer C, Achaval M, Zaro MA, Nigg BM. Human cutaneous sensors on the sole of the foot: Altered sensitivity and recovery time after whole body vibration. Neurosci Lett. 2013;533:581–5. 10.1016/j.neulet.2012.11.036 23201635

[49] Smith DT, Judge S, Malone A, Moynes RC, Conviser J, Skinner JS. Effects of biodensity training and power plate whole-body vibration on strength, balance, and functional independence in older adults. J Aging Phys Act. 2016; 24(1):139–48. 10.1123/japa.2015-0057 26215362

[50] Rittweger J, Beller G, Felsenberg D. Acute physiological effects of exhaustive whole-body vibration exercise in man. Clin Physiol. 2000;20:134–142. 10.1046/j.1365-2281.2000.00238.x 10735981

[51] Shibasaki M. Neural control and mechanisms of eccrine sweating during heat stress and exercise. J Appl Physiol. 2006;100:1692–1701. 10.1152/japplphysiol.01124.2005 16614366

[52] Charkoudian N. Mechanisms and modifiers of reflex induced cutaneous vasodilation and vasoconstriction in humans. J Appl Physiol. 2010;109:1221–1228. 10.1152/japplphysiol.00298.2010 20448028 PMC2963327

[53] Mekjavic IB, Eiken O. Contribution of thermal and nonthermal factors to the regulation of body temperature in humans. J Appl Physiol. 2006;100:2065–2072. 10.1152/japplphysiol.01118.2005 16410380

[54] Pascoe DD, Mercer JD, Weerd L. Physiology of thermal signals. Medical In. Diakides NA, Bronzin JD, editors. Medical Infrared imaging.Boca Raton: CRC Press; 2008.

