# Supplementary material for: Evaluation of the temperature of posterior lower limbs skin during the whole body vibration measured by infrared thermography: Cross-sectional study analysis using linear mixed effect model
Source: PLoS One. 2019 Mar 13;14(3):e0212512. doi: 10.1371/journal.pone.0212512 (PMC6415782; doi:10.1371/journal.pone.0212512)
Supplement: S1 Fig — (PDF) [file pone.0212512.s001.pdf]

RBR-738wng
Avaliação dos efeitos agudos de vibrações geradas em Plataforma oscilante/vibratória em indivíduos jovens e Saudáveis através de parâmetros funcionais

Data de registro: 27 de Julho de 2018 às 17:14

Last Update: 3 de Set. de 2018 às 13:45

Tipo do estudo:

Intervenções

Título científico:

PT-BR	EN
Avaliação dos efeitos agudos de vibrações geradas em Plataforma oscilante/vibratória em indivíduos jovens e Saudáveis através de parâmetros funcionais	Evaluation of the acute effects of vibrations generated in oscillating / vibratory Platform in young and Healthy individuals through functional parameters

Identificação do ensaio

Número do UTN: U1111-1218-2474

Título público:

PT-BR	EN
Efeitos das vibrações geradas em Plataforma oscilante/vibratória em indivíduos jovens e Saudáveis.	Effects of vibrations generated in oscillating / vibratory Platform in young and Healthy individuals

Acrônimo científico:

Acrônimo público:

Identificadores secundários:

CAAE:47933015.1.0000.5259
Órgão emissor: Plataforma Brasil

CEP: 2.612.008
Órgão emissor: Comitê de Ética em Pesquisa do Hospital Universitário Pedro Ernesto/UERJ

Patrocinadores

Patrocinador primário: Universidade do Estado do Rio de Janeiro

Patrocinadores secundários:

Instituição: Universidade do Estado do Rio de Janeiro

Instituição: Hospital Central do Exército

Fontes de apoio financeiro ou material:

Instituição: Conselho Nacional de Desenvimento Científico e Tecnológico

Instituição: Fundação de Amparo à Pesquisa do Estado do Rio de Janeiro - FAPERJ

Condições de saúde

Condições de saúde ou problemas:

PT-BR	EN
jovens saudáveis	healthy young

Descritores gerais para as condições de saúde:

PT-BR	ES	EN
**C23:** Condições patológicas, sinais e sintomas	**C23:** Condiciones patológicas, signos y síntomas	**C23:** Pathological conditions, signs and symptoms

Descritores específicos para as condições de saúde:

PT-BR	ES	EN
**M01.060.116.815:** Adulto Jovem	**M01.060.116.815:** Adulto Joven	**M01.060.116.815:** Young Adult
**M01.390:** Homens	**M01.390:** Hombres	**M01.390:** Men
**M01.975:** Mulheres	**M01.975:** Mujeres	**M01.975:** Women

Intervenções

Categorias das intervenções

Other

Intervenções:

PT-BR

Exercício de vibração de corpo inteiro; exercício isométrico
As sessões de intervenção serão divididas em 6 etapas de uma sessão, nas quais apenas um grupo (n=60) realizará todas as etapas de forma randomizada.
Etapa 1 - Exercício isométrico em posição agachada - 60 participantes realizarão a intervenção em uma única sessão e retornará para outra etapa após um washout de uma semana.
Etapa 2 - Exercício isométrico em flexão de braço - 60 participantes realizarão a intervenção em uma única sessão e retornará para outra etapa após um washout de uma semana.
Etapa 3 - Exercício de vibração de corpo inteiro (30 Hz) em posição agachada - 60 participantes realizarão a intervenção em uma única sessão e retornará para outra

EN

Whole-body vibration exercise; isometric exercise
The intervention sessions will be divided into 6 steps of one session, in which only one group (n = 60) will perform all the steps in a randomized way.
Step 1 - Isometric exercise in crouching position - 60 participants will perform the intervention in a single session and return to another stage after a one-week washout.
Step 2 - Isometric exercise in arm flexion - 60 participants will perform the intervention in a single session and return to another stage after a one-week washout.
Step 3 - Whole body vibration exercise (30 Hz) in a crouched position - 60 participants will perform the intervention in a single session and return to another stage after a one-week washout.
Step 4 - Whole-body vibration exercise (30

etapa após um washout de uma semana.
Etapa 4 - Exercício de vibração de corpo inteiro (30 Hz) em flexão de braço - 60 participantes realizarão a intervenção em uma única sessão e retornará para outra etapa após um washout de uma semana.
Etapa 5 - Exercício de vibração de corpo inteiro (50 Hz) em posição agachada - 60 participantes realizarão a intervenção em uma única sessão e retornará para outra etapa após um washout de uma semana.
Etapa 6 - Exercício de vibração de corpo inteiro (50 Hz) em flexão de braço - 60 participantes realizarão a intervenção em uma única sessão e retornará para outra etapa após um washout de uma semana.
Avaliação Clínica: Os indivíduos terão aferidos massa corporal, estatura, dobras cutâneas e calculados o índice de massa corpórea (IMC) e percentual de gordura. Na história clínica serão valorizados diversos dados obtidos na anamnese, principalmente idade, sexo, doenças associadas, uso de medicamentos e suplementos e prática de exercícios.
Questões éticas: Os indivíduos que farão parte desse trabalho assinarão Termo de Consentimento Livre e Esclarecido antes de qualquer procedimento do estudo. Todos os procedimentos são considerados não invasivos, não trazendo riscos evidentes ao indivíduo.
Aquecimento: Todos os indivíduos, antes de cada sessão, deverão realizar um aquecimento, que consta de 2 minutos de corrida estática, 30 movimentos de polichinelos, 10 agachamentos e 5 movimentos de contração isométrica submáxima com ambas as mãos. Após o aquecimento, os indivíduos terão um repouso de 2 minutos.
Dinanometria: a dinanometria de preensão palmar será realizada conforme preconizado pela American Society of Hand Therapy (ASHT), através de três tentativas, onde o indivíduo irá realizar contração máxima isométrica dos dedos da mão direita durante 5 segundos e com repouso entre as tentativas de 15 segundos. O valor máximo de cada tentativa será utilizado para análise. O dinamômetro manual será o EMG830RF, EMG System, São José dos Campos/SP. Os indivíduos irão realizar este procedimento por 5 vezes, com intervalo de 60 segundos entre os procedimentos.
Caracterização dos parâmetros usados na plataforma oscilante: A plataforma oscilante/vibratória utilizada será do tipo

Hz) in arm flexion - 60 participants will perform the intervention in a single session and return to another stage after a one-week washout.
Step 5 - Whole body vibration exercise (50 Hz) in a crouched position - 60 participants will perform the intervention in a single session and return to another stage after a one-week washout.
Step 6 - Whole body vibration exercise (50 Hz) in arm flexion - 60 participants will perform the intervention in a single session and return to another stage after a one-week washout.
Clinical Evaluation: Individuals will have body mass, height, skinfolds and body mass index (BMI) and fat percentage calculated. In the clinical history will be valued several data obtained in the anamnesis, mainly age, sex, associated diseases, use of medications and supplements and practice of exercises.
Ethical issues: Individuals who will be part of this work will sign a Free and Informed Consent Form prior to any study procedure. All procedures are considered non-invasive, not presenting any obvious risks to the individual.
Heating: All individuals, before each session, should perform a warm-up, consisting of 2 minutes of static running, 30 movements of puppets, 10 squats and 5 movements of submaximal isometric contraction with both hands. After warming up, individuals will have a rest of 2 minutes.
Dynanometry: The palmar gripping dynanometry will be performed as recommended by the American Society of Hand Therapy (ASHT), through three attempts, where the individual will perform isometric maximum contraction of the right hand fingers for 5 seconds and with rest between attempts of 15 seconds. The maximum value of each attempt will be used for analysis. The manual dynamometer will be the EMG830RF, EMG System, São José dos Campos / SP. Individuals will perform this procedure 5 times, with a 60-second interval between procedures.
Characterization of the parameters used in the oscillating platform: The oscillating / vibrating platform used will be of the synchronous triplanar type (Power Plate pro5® - Power Plate International LTD, The Netherlands). Individuals will be exposed to mechanical vibrations in 2 different positions: standing, with knees flexed at 50º and arms hanging, along the body; in an

sincrônica triplanar (Power Plate pro5® - Power Plate International LTD, The Netherlands). Os indivíduos serão expostos às vibrações mecânicas em 2 diferentes posicionamentos: de pé, com joelhos flexionados a 50º e braços pendentes, ao longo do corpo; em posição de flexão de braço, com as mãos apoiadas nas laterais da base da POV, cotovelos levemente fletidos, joelhos apoiados sobre uma plataforma de força e pés descruzados e afastados do solo. Cada exposição terá um tempo um tempo de trabalho de 30 segundos, sendo realizado após 30 segundos de repouso entre cada avaliação de dinamometria, num total de 5 vezes. O investigador irá acompanhar todo procedimento e irá instruir ao indivíduo a relatar qualquer desconforto. Dependendo da situação o procedimento poderá ser encerrado. A frequência utilizada será de 0, 30, 50 Hz e com amplitude de 0 – 0,77 - 1,53 mm (high) respectivamente, com washout de 7 dias. Todos os indivíduos (n=60) deverão realizar todas as etapas de intervenção (cross-over).
Acelerometria: Será realizada a acelerometria durante o contato dos indivíduos com a base da POV em funcionamento. O acelerômetro (EMG830RF, EMG System, São José dos Campos/SP) será posicionado no olécrano direito e sustentado com uso de fita adesiva tipo kinesio tape.
Exercício em barra fixa suspensa: Ao final das avaliações de dinamometria e EVCI, após um repouso de 2 minutos, os indivíduos serão incentivados a manterem-se em contração isométrica durante 30 segundos em uma barra fixa suspensa, com os cotovelos em extensão.
Eletromiografia: será realizado o estudo eletromiográfico dos músculos flexores superficiais e extensores dos dedos no membro superior direito (EMG830RF, EMG System, São José dos Campos/SP). Os eletrodos serão posicionados no ponto médio do ventre muscular dos músculos extensores e flexores dos dedos no antebraço direito. Os dados de rms e frequência mediana serão utilizados para análise. Os indivíduos terão o estudo eletromiográfico registrado durante as avaliações de dinamometria, durante a exposição à vibração e durante o exercício de contração isométrica em barra suspensa.
Durante o EVCI será mensurada a temperatura superficial de membros

arm bending position, with hands resting on the sides of the base of the POV, slightly bent elbows, knees resting on a force platform and feet uncrossed and away from the ground. Each exposure will have a time a working time of 30 seconds, being performed after 30 seconds of rest between each dynamometry evaluation, a total of 5 times. The investigator will follow every procedure and instruct the individual to report any discomfort. Depending on the situation, the procedure may be terminated. The frequency used will be 0, 30, 50 Hz and with amplitude of 0 - 0.77 - 1.53 mm (high) respectively, with washout of 7 days. All individuals (n = 60) should complete all steps of intervention (cross-over).
Accelerometry: Accelerometry will be performed during the contact of the individuals with the base of the functioning POV. The accelerometer (EMG830RF, EMG System, São José dos Campos / SP) will be positioned in the right olecranon and sustained with the use of tape kinesio tape.
Sustained fixed bar exercise: At the end of the dynamometry and EVCI assessments, after a 2-minute rest, individuals will be encouraged to maintain isometric contraction for 30 seconds on a fixed, suspended bar with elbows in extension.
Electromyography: the electromyographic study of the superficial flexor muscles and extensors of the fingers in the right upper limb (EMG830RF, EMG System, São José dos Campos / SP) will be performed. The electrodes will be positioned at the midpoint of the muscle belly of the extensor and flexor muscles of the fingers in the right forearm. The rms and median frequency data will be used for analysis. Individuals will have the electromyographic study recorded during dynamometry assessments, during vibration exposure and during isometric barbell contraction exercise.
During EVCI, lower limb surface temperature will be measured using the FLIR E64 Model Camera (FLIR Systems, Wilsonville, OR, USA).

inferiores utilizando a Câmera Modelo FLIR E64 (FLIR Systems, Wilsonville, OR, EUA).

Descritores para as intervenções:

PT-BR
G11.427.410.698.277: Exercício

ES
G11.427.410.698.277: Ejercicio

Recrutamento

Situação de recrutamento: Recruiting

País de recrutamento
Brazil

Data prevista do primeiro recrutamento: 2015-06-01

Data prevista do último recrutamento: 2022-01-31

Tamanho da amostra alvo:	Gênero para inclusão:	Idade mínima para inclusão:	Idade máxima para inclusão:
60	-	18 -	50 -

Critérios de inclusão:

PT-BR
Indivíduos com idade acima de 18 anos, ambos os sexos, saudáveis

EN
Individuals over 18 years of age, both sexes, healthy

Critérios de exclusão:

PT-BR
diagnóstico de doenças osteo-mio-rticulares; doença neurológica que gere “medo” aos movimentos na plataforma oscilante; doença clínica; a critério do investigador

EN
diagnosis of osteo-myocardial diseases; neurological disease that causes "fear" of the movements on the oscillating platform; clinical disease; at the discretion of the investigator

Tipo do estudo

Desenho do estudo:

PT-BR
Ensaio clínico de prevenção, uni-cego, de braço único.

EN
A one-arm, single-blind clinical prevention trial.

Programa de acesso expandido	Enfoque do estudo	Desenho da intervenção	Número de braços	Tipo de mascaramento	Tipo de alocação	Fase do estudo
Nenhum	Prevention	Single-group	1	Single-blind	Single-arm-study	N/A

Desfechos

Desfechos primários:

PT-BR	EN
Melhora da força de preensão palmar; verificado por meio da dinamometria manual; espera-se um aumento de pelo menos 5% entre o pré e pós intervenção.	Improvement of handgrip strength; verified by means of manual dynamometry; an increase of at least 5% between pre and post intervention is expected.

Desfechos secundários:

PT-BR	EN
Melhora do recrutamento das fibras musculares dos músculos flexores superficias e extensores dos dedos; verificado por meio da eletromiografia de superfície; espera-se um aumento de pelo menos 5% entre o pré e pós intervenção.	Improvement of the recruitment of the muscle fibers of the superficial flexor muscles and extensors of the fingers; verified by surface electromyography; an increase of at least 5% between pre and post intervention is expected.

PT-BR	EN
Mudança na temperatura da pele dos membros inferiores; mensurada por uma câmera termográgica; espera-se analizar as alterações da temperatura da pele durante os exercícios de vibração de corpo inteiro para compreender o efeito das vibrações mecânicas no organismo.	Change in the temperature of the skin of the lower limbs; measured by a thermogagnetic camera; it is expected to analyze changes in skin temperature during whole body vibration exercises to understand the effect of mechanical vibrations on the body.

PT-BR	EN
Melhora da composição corporal; verificada através da bioimpedância; espera-se uma melhora de pelo menos 5% entre o pré e pós intervenção.	Improvement of body composition; verified through bioimpedance; it is expected an improvement of at least 5% between the pre and post intervention.

Contatos

__Contatos para questões públicas__

Nome completo: Mario Bernardo-Filho

Endereço: Boulevard 28 de setembro, 87, fundos, 4º andar, Vila Isabel

Cidade: Rio de Janeiro / Brazil

CEP: 20551-031

Fone: 55-21-28688332

E-mail: bernardofilhom@gmail.com

Filiação: Universidade do Estado do Rio de Janeiro

__Contatos para questões científicas__

Nome completo: Mario Bernardo-Filho

Endereço: Boulevard 28 de setembro, 87, fundos, 4º andar, Vila Isabel

Cidade: Rio de Janeiro / Brazil

CEP: 20551-031

Fone: 55-21-28688332

E-mail: bernardofilhom@gmail.com

Filiação: Universidade do Estado do Rio de Janeiro

Contatos para informação sobre os centros de pesquisa

Nome completo: Mario Bernardo-Filho

Endereço: Boulevard 28 de setembro, 87, fundos,
4º andar, Vila Isabel

Cidade: Rio de Janeiro / Brazil

CEP: 20551-031

Fone: 55-21-28688332

E-mail: bernardofilhom@gmail.com

Filiação: Universidade do Estado do Rio de Janeiro

Links adicionais:

Download no formato ICTRP

Download no formato XML OpenTrials

OpenTrials v1.2
